# Podocyte autophagy is associated with foot process effacement and proteinuria in patients with minimal change nephrotic syndrome

**DOI:** 10.1371/journal.pone.0228337

**Published:** 2020-01-24

**Authors:** Ayu Ogawa-Akiyama, Hitoshi Sugiyama, Masashi Kitagawa, Keiko Tanaka, Yuzuki Kano, Koki Mise, Nozomu Otaka, Katsuyuki Tanabe, Hiroshi Morinaga, Masaru Kinomura, Haruhito A. Uchida, Jun Wada

**Affiliations:** 1 Department of Nephrology, Rheumatology, Endocrinology and Metabolism, Okayama University Graduate School of Medicine, Dentistry and Pharmaceutical Sciences, Okayama, Japan; 2 Department of Human Resource Development of Dialysis Therapy for Kidney Disease, Okayama University Graduate School of Medicine, Dentistry and Pharmaceutical Sciences, Okayama, Japan; 3 Department of Molecular Life Sciences, Tokai University School of Medicine, Kanagawa, Japan; 4 Division of Medical Informatics, Okayama University Hospital, Okayama, Japan; 5 Department of Chronic Kidney Disease and Cardiovascular Disease, Okayama University Graduate School of Medicine, Dentistry and Pharmaceutical Sciences, Okayama, Japan; Kawasaki Ika Daigaku, JAPAN

## Abstract

Autophagy is a cellular mechanism involved in the bulk degradation of proteins and turnover of organelle. Several studies have shown the significance of autophagy of the renal tubular epithelium in rodent models of tubulointerstitial disorder. However, the role of autophagy in the regulation of human glomerular diseases is largely unknown. The current study aimed to demonstrate morphological evidence of autophagy and its association with the ultrastructural changes of podocytes and clinical data in patients with idiopathic nephrotic syndrome, a disease in which patients exhibit podocyte injury. The study population included 95 patients, including patients with glomerular disease (minimal change nephrotic syndrome [MCNS], n = 41; idiopathic membranous nephropathy [IMN], n = 37) and 17 control subjects who underwent percutaneous renal biopsy. The number of autophagic vacuoles and the grade of foot process effacement (FPE) in podocytes were examined by electron microscopy (EM). The relationships among the expression of autophagic vacuoles, the grade of FPE, and the clinical data were determined. Autophagic vacuoles were mainly detected in podocytes by EM. The microtubule-associated protein 1 light chain 3 (LC3)-positive area was co-localized with the Wilms tumor 1 (WT1)-positive area on immunofluorescence microscopy, which suggested that autophagy occurred in the podocytes of patients with MCNS. The number of autophagic vacuoles in the podocytes was significantly correlated with the podocyte FPE score (r = -0.443, p = 0.004), the amount of proteinuria (r = 0.334, p = 0.033), and the level of serum albumin (r = -0.317, p = 0.043) in patients with MCNS. The FPE score was a significant determinant for autophagy after adjusting for the age in a multiple regression analysis in MCNS patients (p = 0.0456). However, such correlations were not observed in patients with IMN or in control subjects. In conclusion, the results indicated that the autophagy of podocytes is associated with FPE and severe proteinuria in patients with MCNS. The mechanisms underlying the activation of autophagy in association with FPE in podocytes should be further investigated in order to elucidate the pathophysiology of MCNS.

## Introduction

Minimal change nephrotic syndrome (MCNS) is one of the most common causes of idiopathic nephrotic syndrome; it is identified in approximately 10–25% of adult patients with the condition [1]. On histological examination, patients with MCNS show no glomerular lesions on light microscopy and no specific findings on fluorescence microscopy; however, electron microscopy (EM) of renal biopsy specimens reveals extensive foot process effacement (FPE) in the glomerular podocytes [2]. Clinically, massive proteinuria is a main diagnostic and therapeutic marker in these patients.

Autophagy is the process through which the bulk degradation of cellular proteins takes place. The cytoplasmic components are enclosed by double-membrane structures known as autophagosomes, which are delivered to lysosomes and then form vacuoles in the cell cytoplasm [3]. The breakdown products in lysosomes are subsequently recycled back to cytoplasm. The *Atg* gene family plays an crucial role in the regulation of cellular autophagy. The p62 gene encodes several proteins that are important for the initiation and maturation of autophagosomes [4–7]. The mammalian target of rapamycin (mTOR) is known to be a key governor of both autophagy and cellular metabolism [8, 9].

The traditional method for observing autophagy within the cell is EM. In the late 1950s, an electron microscopic study demonstrated autophagy in the lysosomes in mammalian cells [10]. At the ultrastructural level, an autophagosome is characterized by a double-membraned structure containing undecomposed cytoplasmic components, which has not fused with a lysosome. Autophagosomes frequently contain intracellular organelles, such as fragments of the endoplasmic reticulum and mitochondria [10].

Besides the physiological role of autophagy in cellular homeostasis, the dysregulation of autophagy may be involved in various disease conditions, such as inflammation, aging, metabolic diseases, neurodegenerative disorders and cancer [11–13]. The DNA of mitochondria that escapes from autophagy leads to Toll-like receptor 9-mediated inflammation and thus induces myocarditis and dilated cardiomyopathy [14]. Recent studies have shown the alteration of autophagy in tubular epithelial cells in renal tubulointerstitial disorder [8, 15–26]. The podocyte-specific knockout of the vacuolar protein sorting defective 34 or the prorenin receptor was associated with severe proteinuria, FPE and the autophagy of numerous podocytes after birth [27–29]. However, little is known about autophagy in glomerular diseases, in particular, in human idiopathic nephrotic syndrome in which alteration of the glomerular podocytes plays a critical role [30–32].

We therefore hypothesized that altered autophagy may be involved in the pathophysiology of MCNS, one of the most common forms of idiopathic nephrotic syndrome. In this study, we investigated the presence of autophagic vacuoles in glomerular podocytes in renal biopsy specimens by EM and further analyzed its association with the degree of FPE in podocytes and the amount of proteinuria, both of which are hallmarks of MCNS.

## Methods

### Subjects

Our study included 95 patients (male, n = 50; female, n = 45) who were admitted to the Unit of Renal Medicine in Okayama University Hospital and underwent percutaneous renal biopsies between February 2007 and January 2015. All procedures in the present study were performed according to national and institutional ethical guidelines for human studies, and guidelines in the Declaration of Helsinki. The ethics committee of Okayama University Hospital approved the study (No. 1607–010). Each patient gave their written informed consent.

### Laboratory measurements

Blood was taken from all subjects in the morning after 12 h of fasting. The serum levels of creatinine, total cholesterol, total protein and albumin were obtained using an automated analyzer (JCA-BM8040; JEOL, Tokyo, Japan). A 24-h urine sample was collected to measure the urinary levels of total protein. The eGFR was calculated as previously described [33].

### Human renal biopsy specimens

Specimens of human renal tissue were obtained by percutaneous renal biopsy. Patients were diagnosed on the basis of clinical symptoms, laboratory data and immunofluorescence, light and EM findings [34]. Control renal tissue specimens were obtained from patients who underwent renal biopsy due to slight asymptomatic proteinuria or hematuria, but in whom glomerular disease was excluded. Each tissue section obtained was routinely evaluated under a light and fluorescence microscope (Olympus, Tokyo, Japan).

### Electron microscopy

Human kidney specimens were observed by EM as described previously [35]. In brief, tissue blocks of kidney pieces were immersed in 2.5% glutaraldehyde for 2 hours at 4°C and subsequently fixed with 1% osmium tetroxide. The blocks were then subjected to dehydration, epon embedding, and ultrathin sectioning. The four serial ultrathin sections were set on a grid and observed under an electron microscope (H7650; Hitachi, Tokyo, Japan). Two ultrastructural types of autophagy are recognized: type I and type II autophagy [36–38]. Type I autophagy is characterized by a condensed ribosome area with a limiting membrane. Type II autophagy is characterized by the aggregation of ribosomes, forming a condensed ribosome area, which always includes numerous aggregated lipid droplets in autophagic vacuoles observed in most of all autophagy. In agreement with previous studies on podocyte autophagy in renal biopsy specimens [36, 37], type I autophagy accounted for <10% of all autophagy and type II autophagy was mainly observed in this study. Thus, we counted autophagic vacuoles (type II autophagy) and the average number of autophagic vacuoles per glomerulus was determined in each subject. A quantitative examination was carried out to count the number of podocyte foot processes per 10 μm of glomerular basement membrane (GBM) in each glomerulus. The mean number of podocyte foot processes was determined as the FPE score, as described previously [39, 40]. Representative electron micrographs used for the evaluation of the podocyte FPE score are shown in **S1 Fig**.

### Immunofluorescence analysis

Renal biopsy specimens were subjected to immunolabelling of microtubule-associated protein 1 light chain 3 (LC3; a homologue of yeast Atg8, which is localized to membranes of autophagosomes) to investigate its cytosolic and autophagosomal distribution. Immunofluorescence staining was performed using 4-μm-thick frozen sections of renal biopsy specimens fixed in cold acetone for 3 min and air dried, as described previously [41]. Double immunofluorescence staining were carried out with rabbit polyclonal anti-LC3 (clone PM036; MBL) diluted 1:50 and mouse monoclonal anti-Wilms tumor 1 (WT1) (clone IR055; DAKO), the latter reacted with the cytoplasm of glomerular podocytes [42, 43]. The sections were blocked in 3% bovine serum albumin for 1 hour at room temperature and then left overnight at 4°C, and stained with the antibodies described above. Next, secondary antibodies conjugated with fluorescein isothiocyanate (FITC) (sc-2012; anti-rabbit IgG, 1:100 dilution, Santa Cruz) and those conjugated with rhodamine (sc-2092; anti-mouse IgG, 1:100 dilution, Santa Cruz) were applied for 1 hour at room temperature. Nuclei were then stained with 4',6-diamidino-2-phenylindole (DAPI) (Life Technologies). Fluorescence images were obtained using an immunofluorescence microscope (BZ-9000; Keyence, Osaka, Japan).

### Statistical analysis

When the data did not follow a normal distribution, they were expressed as the median and interquartile range. A simple linear regression analysis of the data was performed. A multiple regression analysis was performed to determine autophagic vacuoles per glomerulus. P values of < 0.05 were considered to indicate statistical significance. Differences between groups were analyzed using the Mann-Whitney U-test, or the Steel's multiple comparison test as appropriate. The statistical analyses were carried out using the JMP software program (version 11; SAS Institute Inc., Cary, NC, USA).

## Results

### Patient profiles

The baseline characteristics of the study population are shown in **Table 1**. The study population included 95 patients (median age, 55.0 [28.0–69.0] years). Seventeen of the patients were control subjects without any significant clinical or pathological abnormalities (17.9%), 41 patients were diagnosed with MCNS (43.2%), and 37 were diagnosed with idiopathic membranous nephropathy (IMN) (38.9%). At the time of renal biopsy, 28 of 41 patients with MCNS (68.3%) and 9 of 37 patients with IMN (24.3%) had taken corticosteroids and/or immunosuppressants, including cyclosporine for the treatment of glomerular disease. Eighteen of 41 patients with MCNS (43.9%) and 13 of 37 patients with IMN (35.1%) exhibited clinical nephrotic syndrome at the time of renal biopsy.

**Table 1:** The baseline characteristics of the study population.

	**Control** **(n = 17)**	**MCNS** **(n = 41)**	**IMN** **(n = 37)**	**Total** **(n = 95)**
Age (years)	28 (18–39)	40^a^ (23–66)	68^b^^,^ ^d^ (60.5–72.5)	55 (28–69)
Male/Female	7 / 10	21 / 20	22 / 15	48 / 39
eGFR (mL/min/1.73m^2^)	105.2 (83.8–115.8)	77.1^a^ (58.9–105.6)	57.8^b^^,^ ^d^ (47.9–71.4)	72.3 (56.3–98.7)
Serum creatinine (μmol/L)	56.6 (51.3–71.6)	64.5 (55.7–80.9)	80.4 (58.8–97.7)	66.3 (55.7–84.0)
Total Protein (g/L)	74 (71.5–76.5)	49^b^ (42.3–55)	55^b^^,^ ^c^ (49–60.5)	55 (47–67)
Albumin (g/L)	45 (44.5–47)	20^b^ (14.5–29.5)	26^b^^,^ ^c^ (20.5–33.5)	26 (18–38)
Total cholesterol (mmol/L)	4.7 (4.3–5.2)	10.1^b^ (7.1–12.9)	7.0^b^^,^ ^d^ (5.7–8.7)	7.3 (5.5–10.5)
Urinary protein (g/day)	0.1 (0.05–0.2)	3.1^b^ (1.0–6.7)	3.8^b^ (1.6–5.2)	2.4 (0.3–5.0)

eGFR, estimated glomerular filtration rate; MCNS, minimal change nephrotic syndrome; IMN, idiopathic membranous nephropathy.

The clinical data at the time of renal biopsy are expressed as the median value (interquartile range).

^a^: P<0.05

^b^: P<0.01 vs. Control

^c^: P<0.05

^d^: P<0.01 vs. MCNS.

### Morphological evidence of autophagy in renal biopsy specimens

**Fig 1:**
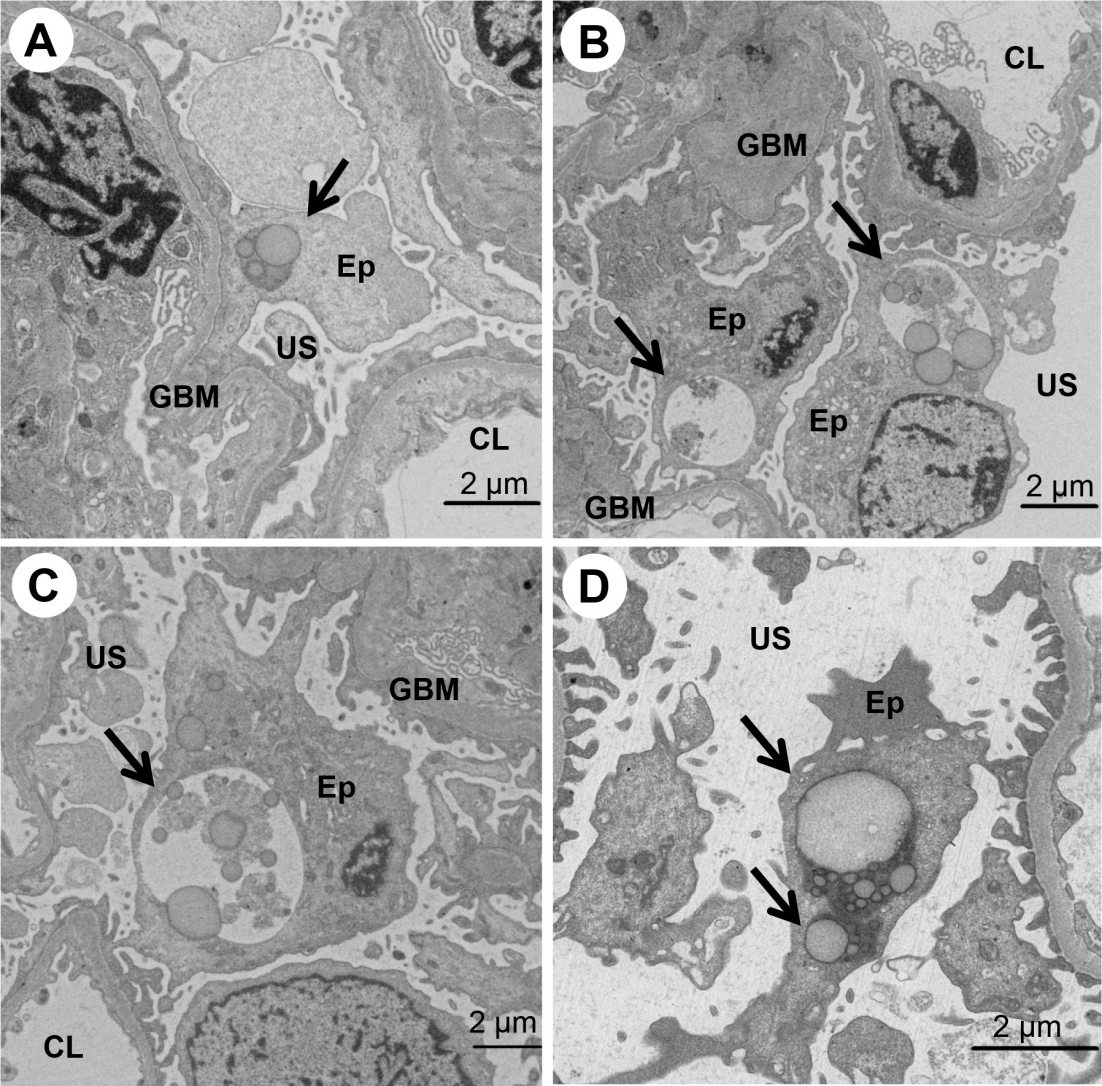
The ultrastructural morphology of autophagy in glomerular podocytes, as observed by electron microscopy. Electron micrographs of a glomerulus in patients with MCNS (A-C, 73-year-old female; D, 19-year-old female) are shown (A-D). A condensed ribosome with limiting membranes (type I autophagy) (A and D) and autophagic vacuoles containing lipid droplets and vacuoles (type II autophagy) (B and C) are indicated by arrows. CL, capillary lumen; Ep, glomerular epithelial cell (podocyte); GBM, glomerular basement membrane; US, urinary space.

**Fig 2:**
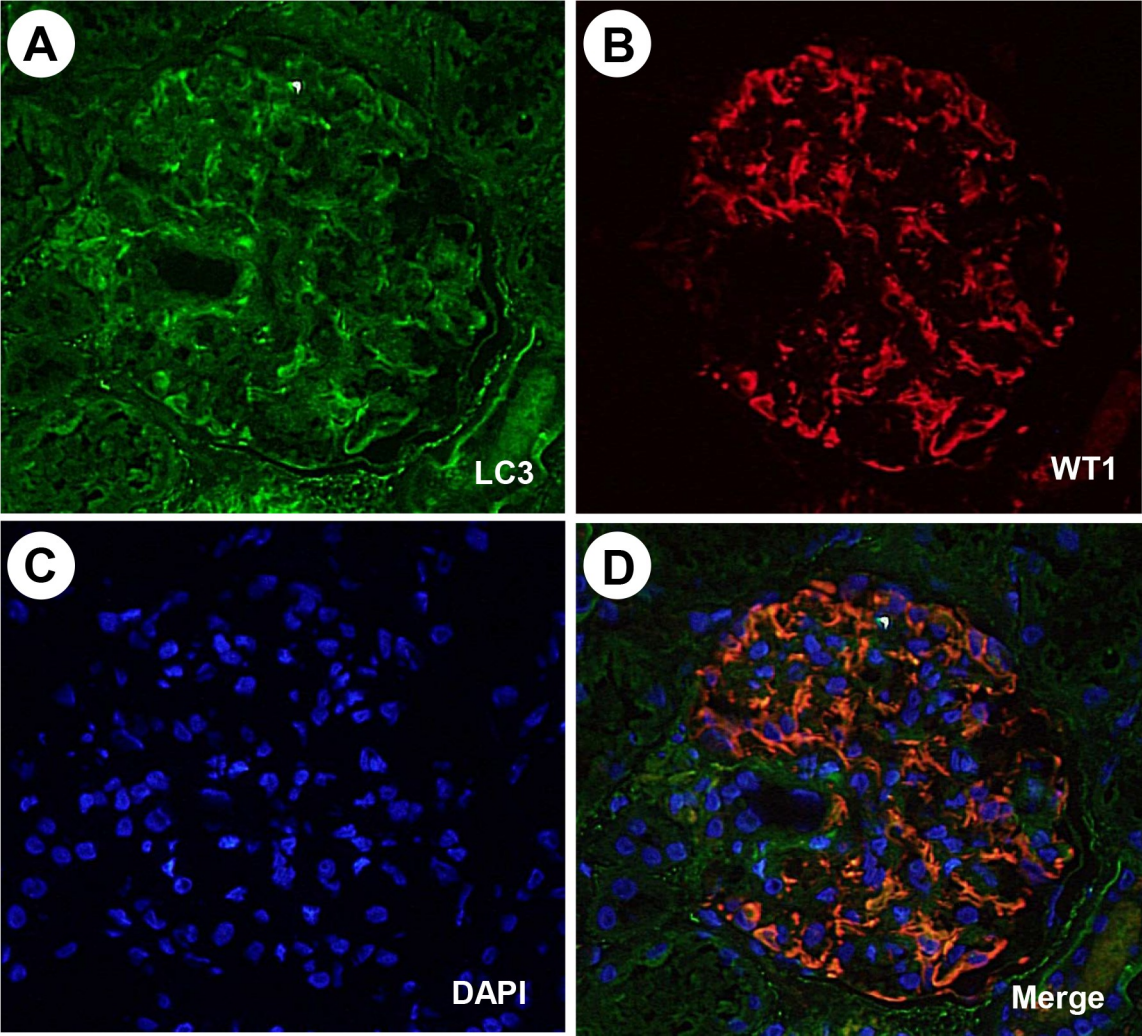
Dual immunolabelling of LC3 and WT1 in the glomerulus of an MCNS patient, as detected by immunofluorescence microscopy (73-year-old, female). Fluorescence micrographs of sections single stained for LC3 with FITC (A), WT1 with rhodamine (B), and dual stained for LC3 and WT1 (D) are shown. The cell nuclei were stained with DAPI (C). Note the LC3-positive area is co-localized with the WT1-positive area in panel D.

Autophagic vacuoles were mainly detected in the cytoplasm of glomerular podocytes, seldom detected in that of glomerular mesangial cells or endothelial cells in renal biopsy specimens observed by EM (**Fig 1**). The localization of LC3, a marker of autophagy, was investigated by co-staining with antibodies to WT1, which are expressed in the cytoplasm of glomerular podocytes in nephrotic syndrome [43]. Most of the LC3-positive podocytes in the glomerulus were co-positive for WT1 (**Fig 2**), suggesting that autophagy mainly occurred in the glomerular podocytes.

### The number of autophagic vacuoles in podocytes was significantly associated with proteinuria, serum albumin, and the podocyte foot process effacement score in patients with MCNS

**Fig 3:**
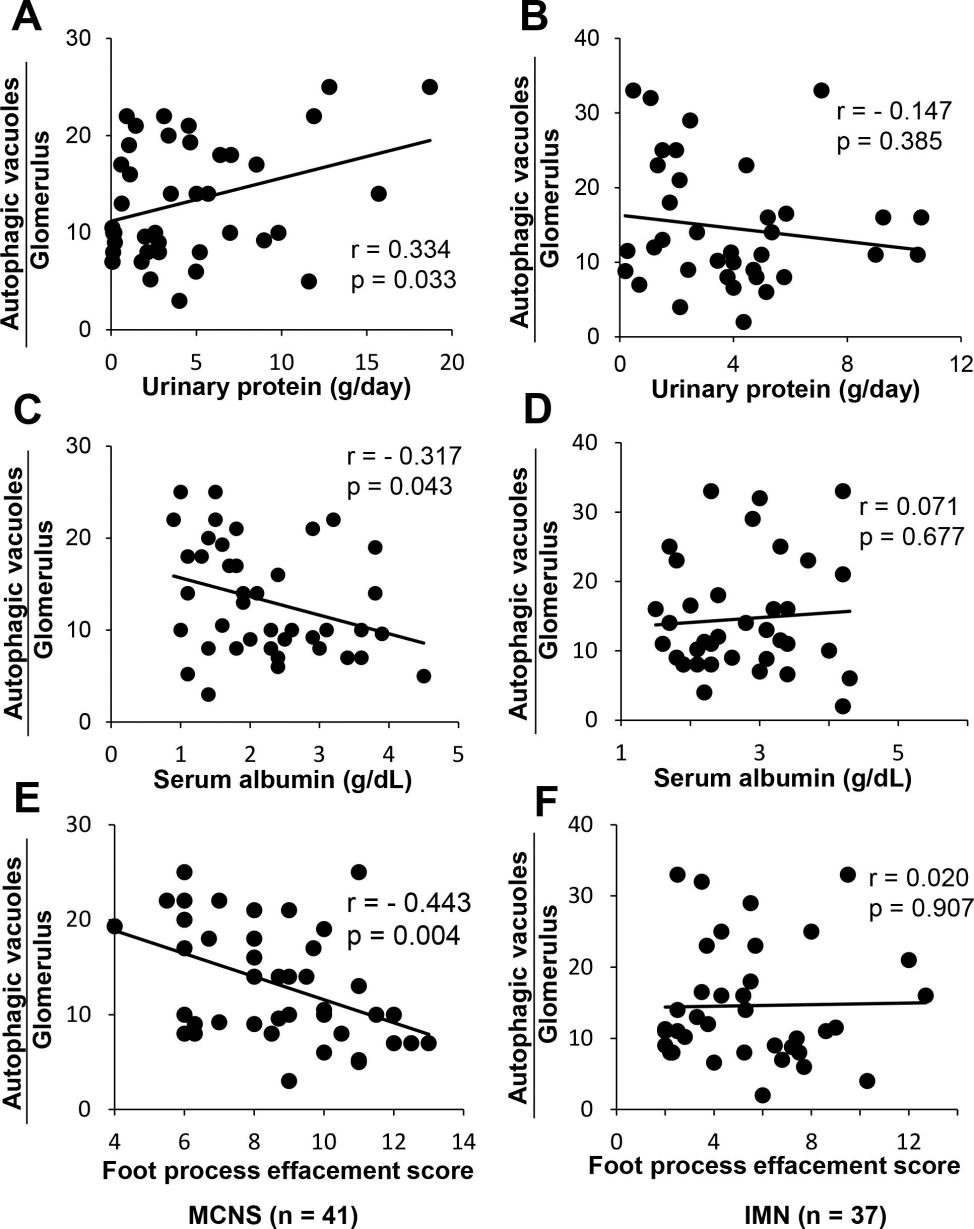
**The relationships between the number of autophagic vacuoles in podocytes and proteinuria, serum albumin, and the foot process effacement score in patients with MCNS (A, C, E) and IMN (B, D, F).** The correlation between the number of the autophagic vacuoles in podocytes and daily urinary protein (g/day) (A, B), serum albumin (g/dL) (C, D) and the podocyte FPE score (E, F). MCNS, minimal change nephrotic syndrome; IMN, idiopathic membranous nephropathy.

We next evaluated the relationships among the number of podocyte autophagic vacuoles, FPE score, and clinical data in patients with MCNS and IMN, who are prone to nephrotic syndrome, and in control subjects (**Table 2**). The number of autophagic vacuoles in podocytes was positively correlated with the amount of urinary protein and was negatively correlated with the serum albumin level in patients with MCNS (**Fig 3A and 3C**), whereas no such correlations were recognized in patients with IMN (**Fig 3B and 3D**). Ultrastructural morphometry revealed that the number of autophagic vacuoles was significantly correlated with the FPE score in podocytes of MCNS patients (**Fig 3E**), while no significant correlations were observed in IMN patients (**Fig 3F**). In control subjects, no significant correlations were observed among the number of autophagic vacuoles and proteinuria, serum albumin, or the FPE score (**S2 Fig**). Since a decline in autophagic activity may play a role in the aging process [11–13], we next examined the relationships between age and the autophagic vacuoles in patients with idiopathic nephrotic syndrome and controls. The number of autophagic vacuoles in podocytes was significantly correlated with age in control subjects and MCNS patients, but not in IMN patients (**S3 Fig**). We further conducted a multiple regression analysis to determine the autophagic vacuoles per glomerulus. After adjusting for the age as a confounding factor, the FPE score was found to be a significant determinant for autophagy in podocytes in patients with MCNS (Table 3). There were no significant determinants for autophagic vacuoles per glomerulus in patients with IMN (S1 Table) or control subjects (S2 Table) after adjusting for the age.

**Table 2:** Evaluation of autophagy and foot process effacement in glomerular podocytes by electron microscopy.

	Control (n = 17)	MCNS (n = 41)	IMN (n = 37)	Total (n = 95)
Autophagic vacuoles per glomerulus	12.0 (7.0–16.0)	10.5 (8.0–18.5)	11.5 (8.4–19.5)	11.5 (8.0–18.0)
Foot process effacement score	16.7 (14.3–19.8)	8.7^a^ (6.5–10.3)	5.3^a^^,^ ^b^ (3.1–7.5)	8.0 (5.5–11.0)

MCNS, minimal change nephrotic syndrome; IMN, idiopathic membranous nephropathy.

Histological data at the time of renal biopsy are expressed as median values (interquartile range).

^a^: P<0.01 vs. Control

^b^: P<0.01 vs. MCNS.

**Table 3:** A multiple regression analysis to determine autophagic vacuoles per glomerulus in MCNS.

Independent variables	β	p-value	model r^2^
Urinary protein (g/day)	0.190368	0.1866	0.3071
Serum albumin (g/dL)	0.006299	0.9731	
Serum creatinine (μmol/L)	-0.192575	0.1709	
Total cholesterol (mmol/L)	0.174846	0.3233	
Foot process effacement score	-0.320560	0.0456*	

Adjusted for age. MCNS, minimal change nephrotic syndrome.

*: P<0.05.

## Discussion

This is the first study to demonstrate the association between autophagy of glomerular podocytes and proteinuria or hypoalbuminemia, both of which are clinical characteristics in patients with MCNS and FPE in podocytes, which is an ultrastructural hallmark in MCNS.

At present, little is known about autophagy in podocytes in human glomerular diseases, in particular in patients with idiopathic nephrotic syndrome. Sato *et al*. demonstrated ultrastructural evidence of autophagy in podocytes in human renal biopsy specimens [36, 37]. They reported that in type I autophagy (approximately 1 μm in diameter), a condensed ribosome area and a few lipid droplets were observed with a limiting membrane originating from mitochondria, while in type II autophagy (3–8 μm in diameter), a condensed ribosome and numerous lipid droplets were observed with a limiting membrane originating from the rough endoplasmic reticulum. Type II autophagy transformed to autophagosomes and autophagic vesicles. Their results suggested that type II autophagy may play a more important role in the clearance of proteins and lipids than type I autophagy. In pediatric patients with IgA nephropathy, which is the most common type of chronic glomerulonephritis [44], the existence of type I autophagy may be associated with a more progressive histopathological class in comparison to type II autophagy [38]. In our study, however, the type I autophagy accounted for <10% of all autophagy, and was much less prevalent than type II autophagy (autophagic vacuoles). Thus, we evaluated the number of autophagic vacuoles alone. The appearance of autophagy should be further investigated in other forms of human glomerular diseases, including lupus nephritis [45] and diabetic nephropathy [26].

The mechanism of interaction between autophagy and FPE in podocytes is largely unknown. In the podocytes of cathepsin D-knockout mice exhibiting proteinuria, the accumulation of podocin, a slit diaphragm (SD) protein, was co-localized with the lysosome-associated membrane glycoprotein 1 and the late endosomal marker Rab7 [46]. The granular structures that accumulated in the podocyte cytoplasm were thus indicated to be autophagosomes and autolysosomes containing degraded podocin, suggesting a degradation of podocin by autophagy, which eventually leads to the disruption of SD and FPE of podocytes. Another candidate mediator involved in the interaction between autophagy and FPE is the Ca^2+^ influx into podocytes. Several studies have shown that Ca^2+^ in the cytoplasm can regulate the cellular process of autophagy [47]. Podocyte FPE involves the dynamic reorganization of actin filaments, a process controlled by the Ca^2+^ influx, as a modulator of the actin cytoskeleton [48].

Focal segmental glomerulosclerosis (FSGS) is another major cause of idiopathic nephrotic syndrome. Patients with the disease show podocyte injury, including FPE, podocyte detachment from the GBM, and podocyte loss, eventually leading to end-stage renal disease with a poorer prognosis than that in MCNS [49]. A recent study showed that the number of autophagosome-positive podocytes in patients with FSGS was significantly lower than that in patients with MCNS [32]. The study showed, using repeat renal biopsies, that decreased podocyte autophagy in MCNS patients was associated with the subsequent progression of MCNS to FSGS. Variants of the apolipoprotein L1 (APOL1) gene in African Americans are known to be related to FSGS [50]. Both soluble urokinase plasminogen activator receptor (suPAR) and APOL1 synergistically induced beta 3 integrin activation in podocytes, resulting in the dysregulation of the actin cytoskeleton, thereby leading to podocyte FPE as well as the formation of dysfunctional autophagosomes [51]. Since we could only obtain a very small number of renal biopsy specimens from patients with FSGS, it was not possible to compare the degree of podocyte autophagy in our study.

Idiopathic membranous nephropathy (IMN) is one of the most frequent causes of nephrotic syndrome in adults, especially in the elderly population [52]. A recent report showed that there is a greater accumulation of LC3-positive vacuoles with the markedly enhanced expression of p62/SQSTM1 protein, a target of autophagy degradation, in the podocytes of IMN patients in comparison to controls, suggesting the impaired degradation of this protein and insufficient autophagy in IMN [31]. Another report demonstrated an increase in Atg3 mRNA in microdissected glomeruli from patients with IMN in comparison to controls [30]. In the current study, we did not recognize a significant increase in the number of autophagic vacuoles in patients with IMN in comparison to controls. The positive correlation of podocyte autophagy with proteinuria, hypoalbuminemia, and FPE, which was observed in patients with MCNS, was not recognized in patients with IMN in this study. Why we noted no relationship between podocyte autophagy and proteinuria in the IMN patients in this study is unknown. We were unable to exclude the possibility that the lower ratio of patients with IMN (24.3%) than that of those with MCNS (68.3%) taking corticosteroids and/or immunosuppressants (including cyclosporine) for the treatment of glomerular disease affected the podocyte autophagy. Clinically, patients with MN exhibit the gradual onset of edema and a slow increase in proteinuria, while those with MCNS exhibit the sudden onset of edema and rapid and massive proteinuria. While this is highly speculative, the difference in the time course of the disease appearance and progression might explain why there was no relationship between podocyte autophagy and proteinuria in IMN patients. Another possible explanation is that IMN is an immune complex disease while MCNS is not. Certain immune complexes can suppress autophagy in glomerular endothelial cells [53]. Immune complexes including specific antigens, such as phospholipase A2 receptor and thrombospondin type-1 domain-containing 7A [54] might affect the interaction of autophagy of podocytes and proteinuria in IMN. Further studies should be performed to investigate the abnormalities in the system of the autophagic degradation of podocytes, such as the expression of p62/SQSTM1 protein, which is reported in other conditions associated with podocyte injury, such as Fabry disease [55, 56].

There are two cellular mechanisms by which misfolded proteins decompose: one is autophagy, which is a lysosome-mediated pathway; the other is the ubiquitin-proteasome system, which is a non-lysosomal and highly specific pathway through which ubiquitinated proteins decompose [57]. Previously published data have reported certain interactions between the two pathways [58]. However, these interactions still remain to be investigated in human podocytes in healthy individuals and in patients with glomerular diseases, such as nephrotic syndrome. In injured podocytes, the counterbalancing of cell survival by autophagy and cell death by apoptosis may play an essential role in the progression of glomerular disease [59–62]. Cross-talk between autophagy and apoptosis via Atg6 homologue beclin 1, which binds to anti-apoptosis bcl-2 in signal transduction, can regulate both autophagy and apoptosis [63]. This interaction in the regulation of podocyte death and survival, which could affect proteinuria and glomerulosclerosis should be further investigated.

The present study is associated with several limitations and strengths that must be kept in mind when understanding the results. First, we did not have data from patients with FSGS, which is a major cause of idiopathic nephrotic syndrome. Since FSGS is less frequent than MCNS and IMN [44], we did not obtain enough samples from patients with FSGS in our study. There are still controversial reports describing a significant decrease in number of autophagic podocytes [32] or an increase in glomerular Atg3 mRNA in relation to autophagy [30] in patients with FSGS in comparison to patients with MCNS. Second, we did not have repeat renal biopsy data, which might have enabled us to obtain causative data that would have allowed us to explore the role of autophagy in disease progression, such as the transition of MCNS to FSGS [32], or the effects of therapeutic interventions (*e*.*g*., rituximab treatment) [64, 65] on autophagy in the podocytes in patients with MCNS. Third, we evaluated the expression of LC3, a marker of autophagic activities as well as disturbed lysosomal recycling. We did not evaluate the autophagy flux [66, 67] in this study, as recommended by the guidelines for the use and interpretation of assays for monitoring autophagy [68]. We were unable to detect the glomerular expression of p62/SQSTM1 protein, which is degraded by autophagy and therefore serves as a marker of impaired autophagy (unpublished observation). Fourth, we included patients who were already undergoing treatment with corticosteroids and/or immunosuppressants at the time of renal biopsy; thus, we could not exclude the possibility that these treatments affected the autophagy status in podocytes. Fifth, this study contained an insufficient number of controls who were age-matched with patients with MCNS, so a sufficient number of age-matched controls should be accumulated in the future.

In conclusion, we have demonstrated that there were significant associations between the autophagy of glomerular podocytes and proteinuria, hypoalbuminemia, and FPE in podocytes in patients with MCNS. Such correlations were not recognized in patients with IMN, another form of proteinuric glomerular disease. Further investigations should be performed to clarify the autophagic flux and autophagic activities in podocytes in MCNS. The precise mechanism of molecular interaction between autophagy and FPE in the podocytes should be examined, since modulation of this interaction may have novel therapeutic applications in the treatment of MCNS.

## Supporting information

S1 Table A multiple regression analysis to determine autophagic vacuoles per glomerulus in IMN. (DOCX)

S2 Table A multiple regression analysis to determine autophagic vacuoles per glomerulus in control. (DOCX)

S1 Fig Representative electron micrographs used for the evaluation of foot process effacement in glomerular podocytes. Panel A shows an FPE score of ≥15 (15-years-old, male, control). Panel B shows an FPE score of ≥8 to <15 (19-year-old, female, MCNS). Panel C shows an FPE score of <8 (26-year-old, male, MCNS).
(TIF)

S2 Fig **The relationship between the number of autophagic vacuoles and urinary protein (g/day) (A), serum albumin (mg/dL) (B), and the foot process effacement score (C) in control subjects (n = 17).** There were no significant correlations between the number of autophagic vacuoles and any of the parameters.
(TIF)

S3 Fig **The relationship between the number of autophagic vacuoles and age in control subjects (A), MCNS patients (B) and IMN patients (C).** The number of autophagic vacuoles were significantly correlated with age in the control subjects (n = 17) (A) and MCNS patients (n = 41) (B), but not in the patients with IMN (n = 37) (C).
(TIF)
