# Supplementary material for: Podocyte autophagy is associated with foot process effacement and proteinuria in patients with minimal change nephrotic syndrome
Source: PLoS One. 2020 Jan 24;15(1):e0228337. doi: 10.1371/journal.pone.0228337 (PMC6980606; doi:10.1371/journal.pone.0228337)
Supplement: S1 Table — (DOCX) [file pone.0228337.s001.docx]

**S1 Table. A multiple regression analysis to determine autophagic vacuoles per glomerulus in IMN.**

| **Independent variables** | **β** | **p-value** | **model r^2^** |
| --- | --- | --- | --- |
| Urinary protein (g/day) | -0.060404 | 0.7861 | -0.069 |
| Serum albumin (g/dL) | -0.151368 | 0.6200 |  |
| Serum creatinine (μmol/L) | -0.286720 | 0.1966 |  |
| Total cholesterol (mmol/L) | -0.280200 | 0.2345 |  |
| Foot process effacement score | -0.036427 | 0.8703 |  |

Adjusted for age. IMN, idiopathic membranous nephropathy.
