# Supplementary material for: Podocyte autophagy is associated with foot process effacement and proteinuria in patients with minimal change nephrotic syndrome
Source: PLoS One. 2020 Jan 24;15(1):e0228337. doi: 10.1371/journal.pone.0228337 (PMC6980606; doi:10.1371/journal.pone.0228337)
Supplement: S2 Table — (DOCX) [file pone.0228337.s002.docx]

**S2 Table. A multiple regression analysis to determine autophagic vacuoles per glomerulus in control.**

| **Independent variables** | **β** | **p-value** | **model r^2^** |
| --- | --- | --- | --- |
| Urinary protein (g/day) | 0.090334 | 0.8515 | 0.3680 |
| Serum albumin (g/dL) | 0.384002 | 0.4048 |  |
| Serum creatinine (μmol/L) | -0.100927 | 0.7300 |  |
| Total cholesterol (mmol/L) | -0.212501 | 0.5200 |  |
| Foot process effacement score | 0.172262 | 0.5777 |  |

Adjusted for age.
