# Supplementary figures and images for: Podocyte autophagy is associated with foot process effacement and proteinuria in patients with minimal change nephrotic syndrome
Source: PLoS One. 2020 Jan 24;15(1):e0228337. doi: 10.1371/journal.pone.0228337 (PMC6980606; doi:10.1371/journal.pone.0228337)

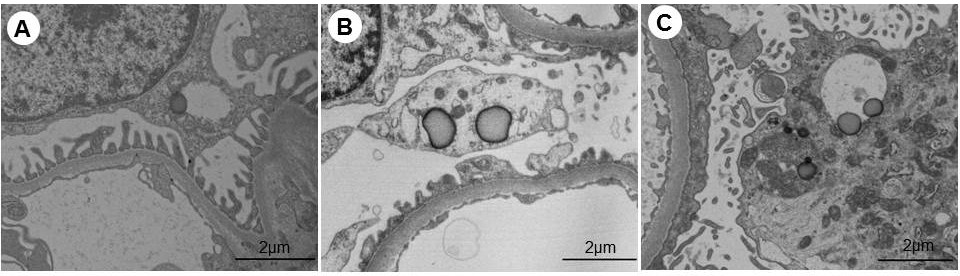

Supplement: S1 Fig — Panel A shows an FPE score of ≥15 (15-years-old, male, control). Panel B shows an FPE score of ≥8 to <15 (19-year-old, female, MCNS). Panel C shows an FPE score of <8 (26-year-old, male, MCNS). (TIF) [file pone.0228337.s003.tif]

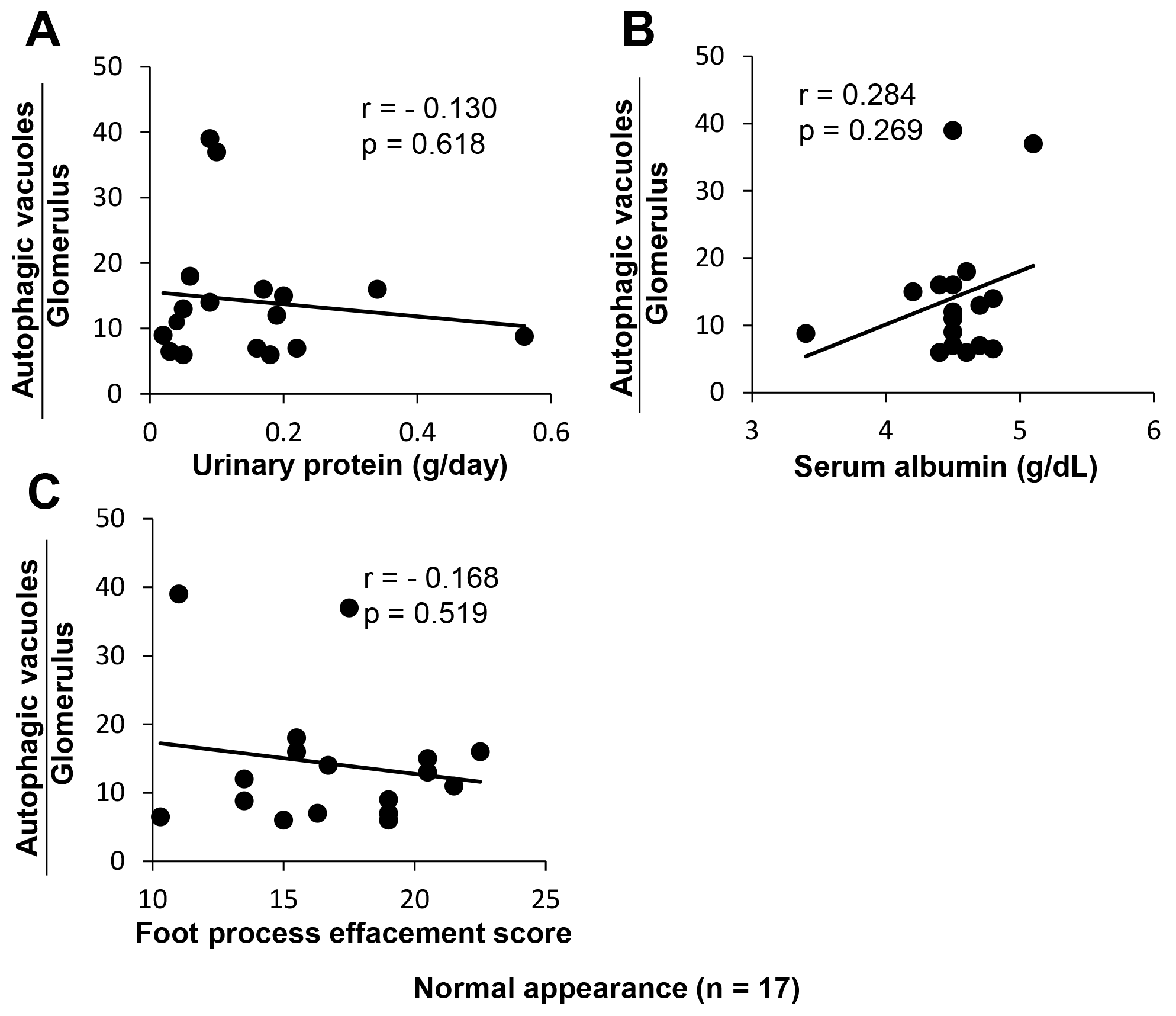

Supplement: S2 Fig — The relationship between the number of autophagic vacuoles and urinary protein (g/day) (A), serum albumin (mg/dL) (B), and the foot process effacement score (C) in control subjects (n = 17). There were no significant correlations between the number of autophagic vacuoles and any of the parameters. (TIF) [file pone.0228337.s004.tif]

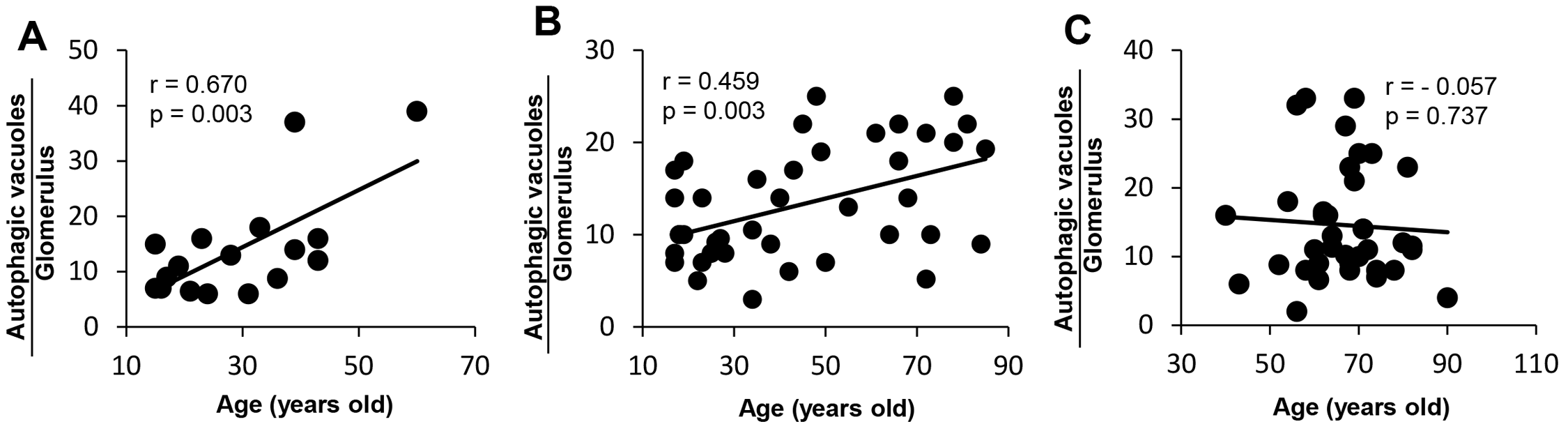

Supplement: S3 Fig — The relationship between the number of autophagic vacuoles and age in control subjects (A), MCNS patients (B) and IMN patients (C). The number of autophagic vacuoles were significantly correlated with age in the control subjects (n = 17) (A) and MCNS patients (n = 41) (B), but not in the patients with IMN (n = 37) (C). (TIF) [file pone.0228337.s005.tif]
